# Long COVID: health symptoms, impact on health-related quality of life and mapping to disability weights: Juanita Haagsma

**DOI:** 10.1093/eurpub/ckac129.139

**Published:** 2022-10-25

**Authors:** J Haagsma

**Affiliations:** Public Health, Erasmus MC, Rotterdam, Netherlands

## Abstract

**Introduction:**

Previous studies indicated that a significant share of COVID-19 patients experiences long lasting health complaints; a condition also referred to as “long COVID”. In order to assess the long term burden of disease of COVID-19, including long COVID, information is needed on health symptoms, health-related quality of life and duration of symptoms of long COVID patients. Therefore, the aim of this study was to assess health symptoms and health-related quality of life of long COVID in the general population of the Netherlands.

**Methods:**

A total of 33,903 COVID-19 patients from the region South-Holland South (the Netherlands) whom tested positive between June 2020 and May 2021 at the municipal health services were invited to complete a web-based questionnaire on the presence, nature and consequences of long COVID and health-related quality of life, measured with the EQ-5D-5L.

**Results:**

In total, 3,768 participants who completed the questionnaire experienced long COVID. Most commonly reporting symptoms were reduced physical condition (65.5%), fatigue (59.6%), problems concentrating (49.6%), loss of smell (41.2%) and shortness of breath (37.8%). 648 participants indicated that they experienced long COVID symptoms for 5 months or longer. Preliminary analysis showed that increasing number of symptoms was associated with a decrease in health-related quality of life.

**Discussion and conclusions:**

Long COVID covers a range of health symptoms of varying severity and duration. This complicates the calculation of the non-fatal burden of disease of COVID-19, particularly because mapping of long COVID to existing disability weights is hampered.

